# Combined Modulation of Ceramide and S1P Pathways Induces Strong Cytotoxicity in SH-SY5Y Neuroblastoma Cells

**DOI:** 10.3390/ijms27188126

**Published:** 2026-09-12

**Authors:** Celal Ozbek Cakir, Canan Vejselova Sezer, Mustafa Cengiz, Hatice Mehtap Kutlu

**Affiliations:** 1 Department of Neurosurgery, Afyon Park Hayat Hospital, Afyon 03100, Türkiye; celal_ozbek@yahoo.com; 2 Department of Biology, Kutahya Dumlupinar University, Kütahya 43000, Türkiye; 3 Department of Mathematics and Physical Sciences Education, Siirt University, Siirt 56100, Türkiye; m.cengiz@siirt.edu.tr; 4 Department of Biology, Eskisehir Technical University, Eskisehir 26470, Türkiye; hmkutlu@eskisehir.edu.tr

**Keywords:** neuroblastoma, fingolimod, ceranib-2, carmofur, ceramide, apoptosis

## Abstract

Neuroblastoma is among the most aggressive pediatric cancers, and resistance to standard therapies highlights the need for alternative strategies targeting lipid metabolism and apoptosis-associated pathways. This study aimed to investigate the cytotoxic and apoptotic effects of fingolimod, ceranib-2, and carmofur, administered individually and in combination, in SH-SY5Y neuroblastoma cells, with particular emphasis on ceramide metabolism. Cytotoxicity was evaluated using the MTT assay, and morphological alterations were assessed by confocal microscopy. Colony-forming ability was examined using a soft agar assay, apoptosis-associated responses were evaluated by Annexin-V staining and caspase 3/7 activation analysis, and intracellular ceramide levels were determined using an ELISA-based method. The results showed that all three compounds reduced cell viability in a concentration-dependent manner and decreased clonogenic survival. The triple combination produced the most pronounced changes in apoptosis-associated parameters and showed marked inhibition of colony formation, although fingolimod alone exhibited the lowest IC_50_ value and therefore remained the most potent treatment on an IC_50_ basis. Confocal microscopy revealed prominent apoptosis-associated morphological alterations, including chromatin condensation, nuclear fragmentation, and cytoskeletal disruption, particularly in the triple-combination group. Annexin-V and caspase 3/7 analyses showed increased apoptotic cell populations, with the highest levels observed following triple-combination treatment. Intracellular ceramide levels were also significantly elevated, particularly in cells treated with ceranib-2 and the triple combination. Collectively, these findings indicate that simultaneous modulation of ceramide- and S1P-associated pathways influences apoptosis-associated responses, clonogenic growth, and intracellular ceramide levels in SH-SY5Y cells. However, because the study was conducted in a single neuroblastoma cell line and did not include comprehensive dose-by-time or formal combination-interaction analyses, the findings should be regarded as preliminary in vitro observations. Further validation in genetically distinct neuroblastoma models, together with systematic dose–response and time–response studies, will be required to determine the reproducibility, biological significance, and broader therapeutic relevance of this approach.

## 1. Introduction

Neuroblastoma is an aggressive neoplasm arising from embryonic cells of the peripheral nervous system, primarily affecting children [1]. Current clinical management methods for neuroblastoma include chemotherapy, radiotherapy, surgery, immunotherapy, and gene therapy [2]. The development of resistance to chemotherapeutic drugs such as cisplatin, carboplatin, etoposide, vincristine, and doxorubicin, together with significant organ toxicities, presents major clinical challenges in treatment. As a result, research is intensifying to develop novel therapeutic approaches that are more effective and have reduced toxicity for neuroblastoma treatment [1,2,3].

The SH-SY5Y human neuroblastoma cell line is widely used as an in vitro model for investigating neuronal and cancer-related cellular mechanisms and for the preliminary assessment of pharmacological compounds [4,5]. SH-SY5Y cells retain catecholaminergic neuronal characteristics and functional apoptotic machinery, making them useful for investigating sphingolipid-associated cellular responses. However, neuroblastoma is a molecularly and phenotypically heterogeneous malignancy, and individual cell lines represent only part of this biological diversity [6]. MYCN amplification is one of the major molecular features associated with high-risk neuroblastoma and is closely related to aggressive tumor behavior and unfavorable clinical outcome [7,8]. Therefore, findings obtained from a single cell line should be interpreted within the context of that specific experimental model. Accordingly, the present study should be regarded as an initial evaluation in SH-SY5Y cells, and validation in additional genetically distinct neuroblastoma cell lines, including MYCN-amplified models, will be necessary to determine the broader applicability of the observed effects. The balance between life and death in neuronal cells is regulated by sphingolipid signaling pathways, primarily through lipid metabolism. Sphingolipids are essential and complex lipids found predominantly in the cell membranes of eukaryotic cells, particularly within the myelin and neuronal membranes of the central nervous system (CNS). These lipids are crucial constituents influencing the structure and fluidity of the cell membrane, thereby modulating important biological processes such as signal transmission, cell differentiation, apoptosis, and autophagy. The metabolic pathways of sphingolipids are complex and conserved, producing several metabolites. Metabolites including ceramide (Cer) and sphingosine-1-phosphate (S1P) play crucial roles in central nervous system signalling and affect neurodevelopment, myelination, and synaptic plasticity. Therefore, disruption of sphingolipid metabolism is closely associated with brain diseases [9].

Ceramide species are generated through distinct ceramide synthases and may exert different biological effects depending on their acyl-chain composition [10,11,12,13,14]. In neuronal and cancer-related cellular contexts, alterations in ceramide metabolism have been associated with mitochondrial dysfunction, oxidative stress, autophagy, and cell death signaling [13,14,15]. These observations highlight the biological relevance of ceramide metabolism while also emphasizing the complexity of species-specific ceramide functions. 

Acid ceramidase (ASAH1), an essential enzyme in sphingolipid metabolism, initiates signaling pathways that promote cell proliferation and survival by converting ceramide into sphingosine. Inhibition of ASAH1 may lead to the accumulation of intracellular ceramide, thereby triggering apoptotic and stress-induced cell death processes [16]. Consequently, ASAH1 is considered a significant therapeutic target, particularly for oncological and neurodegenerative disorders. Numerous small molecules influence sphingolipid metabolism at various stages. Ceranib-2, Carmofur, and Fingolimod (FTY720) are compounds documented in the recent literature, and their anticancer efficacy is currently under investigation. Ceranib-2 and carmofur are classified as acid ceramidase inhibitors, whereas fingolimod is described as a small molecule that affects cellular viability, inflammation, and mitochondrial signaling through sphingosine-1-phosphate receptors [17,18,19]. These compounds are known for their cytotoxic or antiproliferative effects in various cancer cell lines and several cellular models [20,21,22,23]. The rationale for combining two acid ceramidase inhibitors with fingolimod is that the three compounds act on distinct nodes of the same ceramide/sphingosine-1-phosphate axis: ceranib-2 and carmofur each limit the conversion of ceramide to sphingosine and are therefore expected to raise intracellular ceramide, whereas fingolimod attenuates the pro-survival S1P signalling that opposes ceramide-driven apoptosis. SH-SY5Y cells have been widely used to investigate cellular proliferation and cytotoxic responses in neurobiological experimental settings [24], while the biological relevance of sphingosine-1-phosphate signalling and its modulation in therapeutic contexts has been extensively described [25]. Targeting the pathway simultaneously at more than one point may shift the ceramide/S1P balance towards ceramide accumulation more effectively than blocking a single enzyme [26]. The cytotoxic effects of such a triple combination of sphingolipid cascade-targeting small molecules in the SH-SY5Y cell line have not previously been assessed. This study evaluates, for the first time in this model, the cytotoxic capacity and concentration–response relationship of the three compounds applied individually and in combination. In the present design, the compounds were compared as single agents and as the triple combination; the intermediate two-compound combinations were not tested, and this is noted among the study limitations.

Ceramide has been demonstrated to trigger apoptosis in neoplastic cells. Ceramide can be synthesised via sphingomyelinase-mediated or de novo pathways. Sphingomyelinases facilitate the release of ceramide by hydrolysing sphingomyelin in biological membranes. Ceramide-mediated cell death in tumour cells is initiated by various stimuli, such as CD95, TNF receptor, DR5, gamma irradiation, cytotoxic agents, ultraviolet light, bacteria, viruses, and disruption of the cell–matrix interface. This study aimed to evaluate the effects of fingolimod, carmofur, and ceranib-2, administered individually and in combination, on cell viability and apoptosis-associated responses in SH-SY5Y neuroblastoma cells, and to provide an initial in vitro assessment of the biological effects of simultaneously targeting different components of sphingolipid metabolism.

## 2. Results

### 2.1. MTT Findings

**Figure 1:**
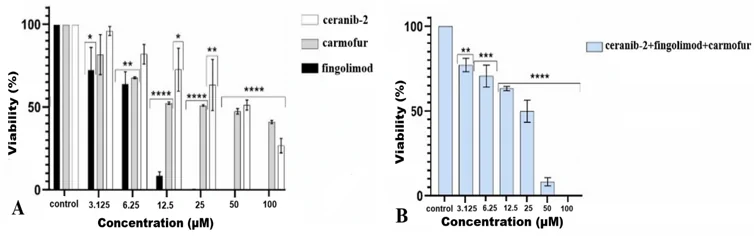
Concentration-dependent effects of fingolimod, ceranib-2, carmofur, and their triple combination on SH-SY5Y cell viability after 24 h, as determined by the MTT assay. (**A**) Fingolimod, ceranib-2, and carmofur (* *p* < 0.0258, ** *p* < 0.003, **** *p* < 0.0001); (**B**) triple combination (** *p* < 0.0033, *** *p* < 0.0004, **** *p* < 0.0001).

The IC_50_ values determined from the application of the active compounds individually to SH-SY5Y cells over a 24-h period were 52.87 ± 1.76 µM, 33.41 ± 1.21 µM and 7.84 ± 0.44 µM for ceranib-2, carmofur and fingolimod, respectively, and 24.85 ± 1.12 µM for the triple combination. For the triple combination, the IC_50_ value represents the total concentration of the three compounds administered at a 1:1:1 ratio, corresponding to approximately 8.28 µM of each compound. On a single-agent basis, fingolimod was the most potent of the three compounds, with the lowest IC_50_. Viability and proliferation decreased with increasing concentrations of the administered drugs, indicating concentration-dependent cytotoxicity. The greatest reduction in viability occurred at the highest concentration of each compound to which the cells were exposed (Figure 1).

### 2.2. Confocal Microscopy Findings

**Figure 2:**
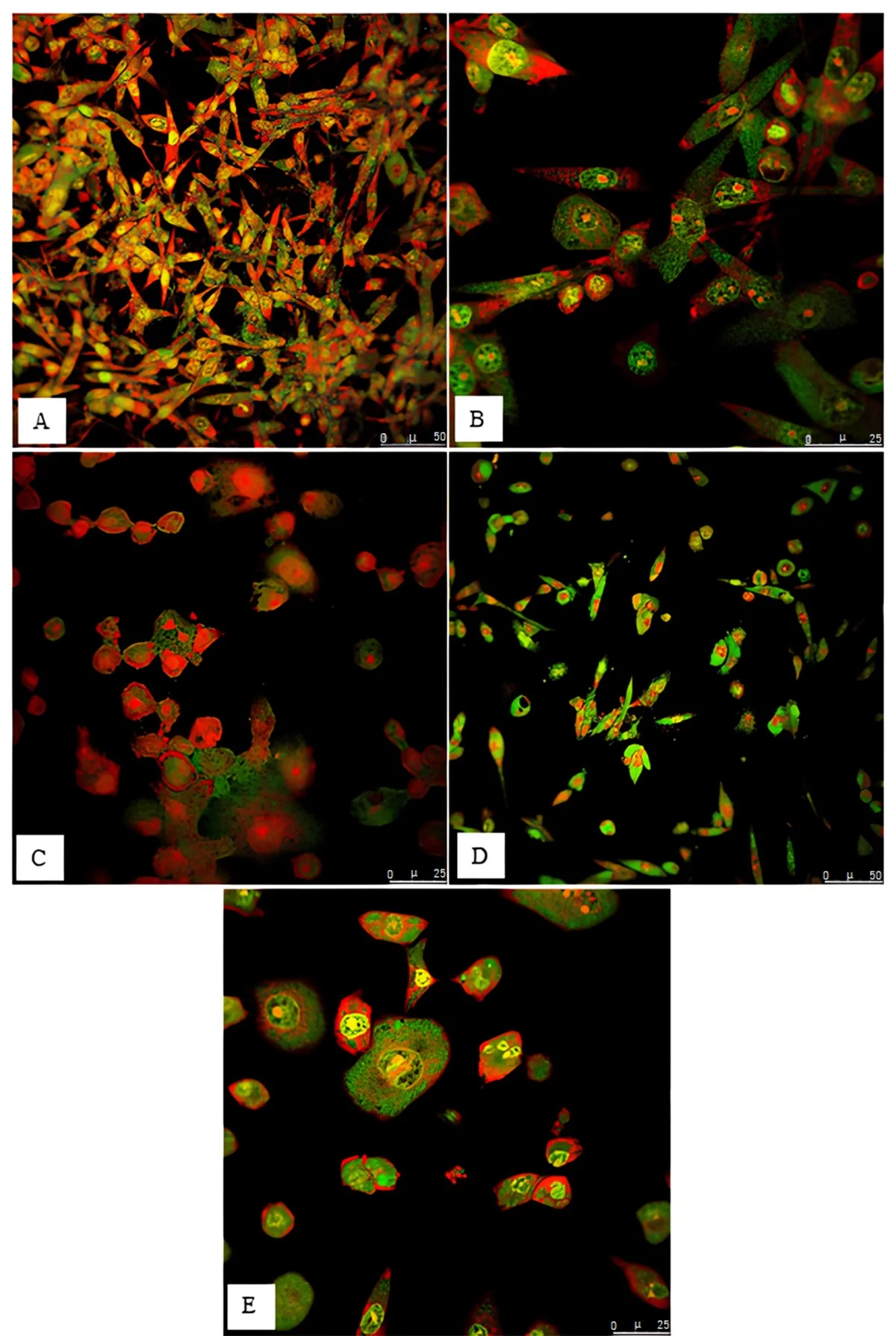
Confocal micrographs of SH-SY5Y cells treated with IC_50_ concentrations. (**A**) Control; (**B**) fingolimod; (**C**) ceranib-2; (**D**) carmofur; (**E**) combination. Scale bars = 50 µm in panels (**A**,**D**), and 25 µm in panels (**B**,**C**,**E**). Images are representative of three independent experiments (*n* = 3), with 10 fields examined per condition.

Control group cells displayed normal morphology without alterations (Figure 2A). Cells treated with the IC_50_ concentration of fingolimod showed nuclear perforations, cytoskeletal disruption, and a rounded morphology (Figure 2B). In SH-SY5Y cells exposed to ceranib-2, chromatin condensation, cytoskeletal disruption, membrane blebbing, cell rounding, and shrinkage were observed (Figure 2C). Cells treated with carmofur exhibited morphological changes including shrinkage, rounding, and cytoskeletal disintegration (Figure 2D). In SH-SY5Y cells treated with the combination of fingolimod, ceranib-2, and carmofur, prominent nuclear fragmentation, horseshoe-shaped nuclei, shrinkage, and cytoskeletal disruption were observed (Figure 2E).

### 2.3. Soft Agar Colony Formation Assay Findings

**Figure 3:**
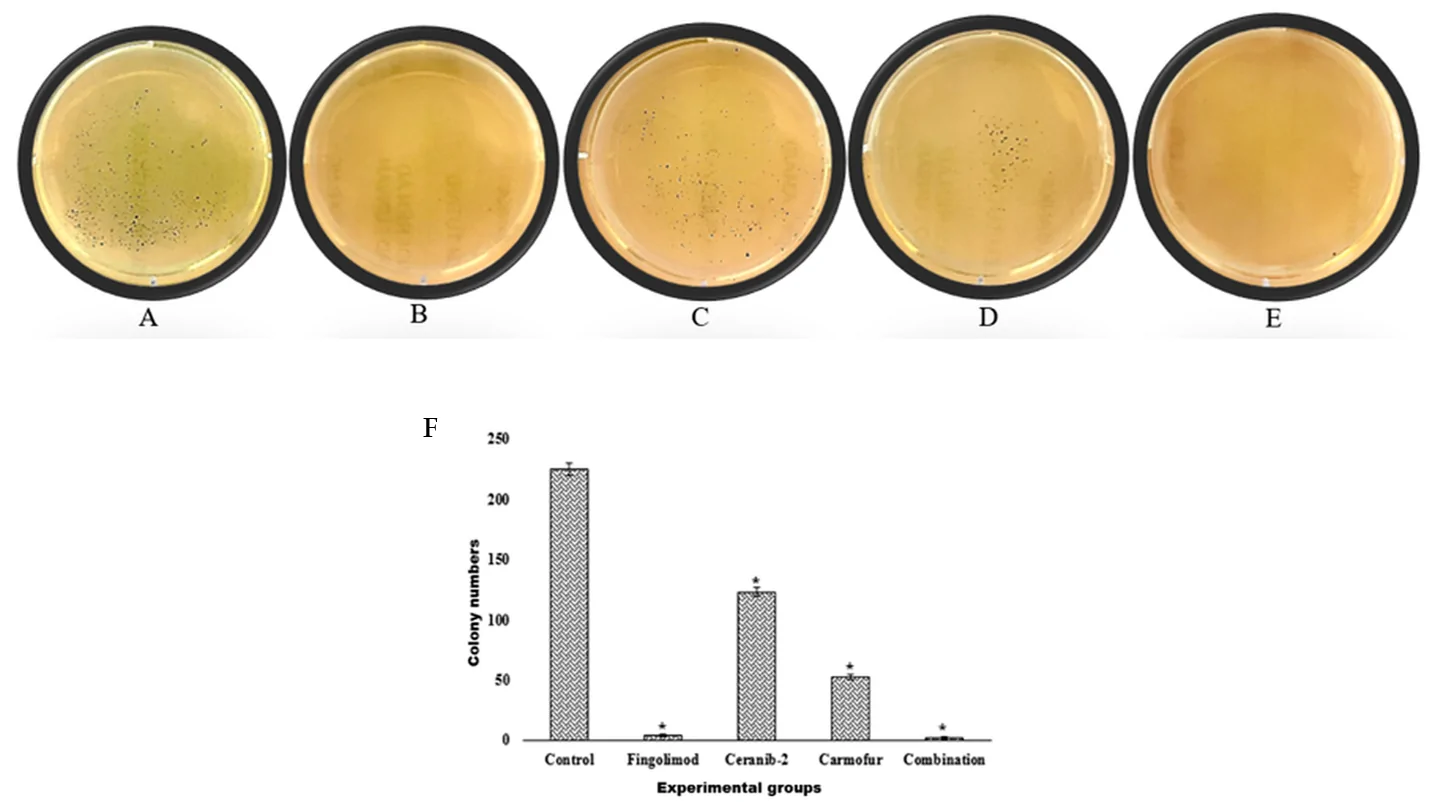
Soft agar colony formation of SH-SY5Y cells following treatment with fingolimod, ceranib-2, carmofur, and their triple combination. (**A**) Control; (**B**) fingolimod; (**C**) ceranib-2; (**D**) carmofur; (**E**) combination; (**F**) quantification of colony numbers. Data are presented as mean ± SD from three independent experiments (*n* = 3), with three technical replicates per condition. Statistical comparisons were performed using one-way ANOVA followed by Tukey’s post hoc test. * *p* < 0.05 versus the control group.

The colony counts observed in SH-SY5Y cells were 225 ± 5, 4 ± 1, 123 ± 4, 52 ± 2, and 2 ± 1 for the control, fingolimod, ceranib-2, carmofur, and triple combination groups, respectively. A significant reduction in colony count was found compared to the control group for each applied agent. Colony inhibition was most pronounced in cells treated with the triple combination. The least pronounced inhibition among the treated groups was observed with ceranib-2. Marked colony inhibition was noted in cells treated with fingolimod (*p* < 0.0023) and carmofur (*p* < 0.0012). The highest inhibition was recorded in cells treated with triple combination (*p* < 0.001) (Figure 3).

### 2.4. Annexin-V Findings

**Figure 4:**
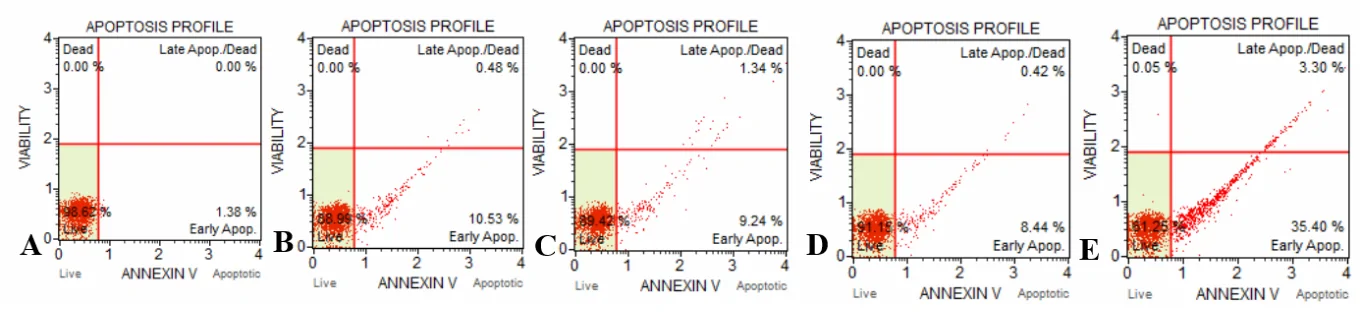
Annexin-V apoptosis profiles of SH-SY5Y cells following treatment with fingolimod, ceranib-2, carmofur, and their triple combination at IC_50_ concentrations. (**A**) Control; (**B**) fingolimod; (**C**) ceranib-2; (**D**) carmofur; (**E**) combination. Representative profiles from three independent experiments.

The viable cell percentages observed in the profiles for the control, fingolimod, ceranib-2, carmofur, and combination groups were 98.62 ± 2.26%, 88.99 ± 1.31%, 89.42 ± 0.96%, 91.15 ± 2.02%, and 61.25 ± 0.95%, respectively. The total apoptotic fractions, comprising early- and late-apoptotic cells, were 1.38%, 11.01%, 10.58%, 8.86%, and 38.70% for the control, fingolimod, ceranib-2, carmofur, and combination groups, respectively. The combination group had the highest percentage of cells in early apoptosis at 35.40%, while the control group had the lowest at 1.38%. In SH-SY5Y cells treated with fingolimod, ceranib-2, and carmofur, the respective early apoptotic cell percentages were 10.53%, 9.24%, and 8.44%. The proportions of cells in late apoptosis were minimal across all groups (Figure 4).

### 2.5. Caspase 3/7 Activation Analysis Findings

**Figure 5:**
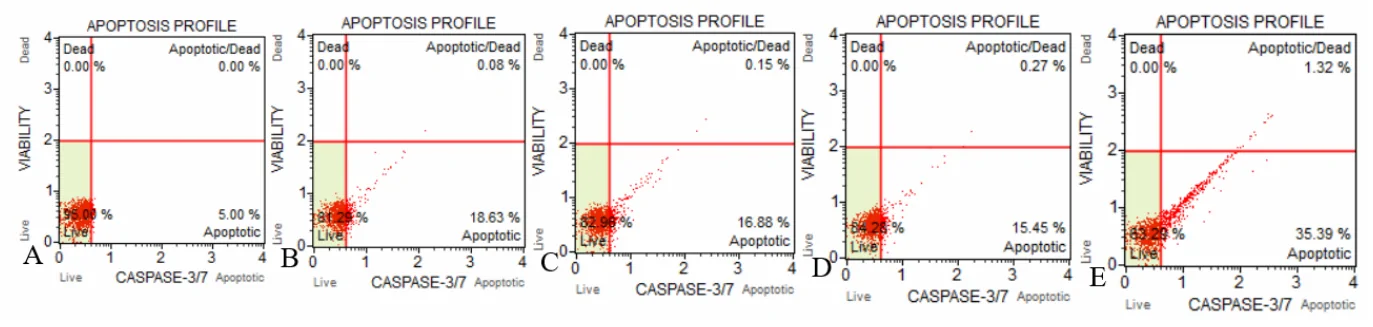
Caspase 3/7 activation profiles of SH-SY5Y cells following treatment with fingolimod, ceranib-2, carmofur, and their triple combination at IC_50_ concentrations. (**A**) Control; (**B**) fingolimod; (**C**) ceranib-2; (**D**) carmofur; (**E**) combination. Representative profiles from three independent experiments.

The percentages of live cells observed in the caspase 3/7 activation analysis for the control, fingolimod, ceranib-2, carmofur, and combination groups were 95.00 ± 3.12%, 81.29 ± 1.13%, 82.96 ± 1.34%, 84.28 ± 2.45%, and 63.29 ± 1.97%, respectively. The total apoptosis-associated fractions, including both apoptotic and apoptotic/dead cells, were 18.71%, 17.03%, 15.72%, and 36.71% for fingolimod, ceranib-2, carmofur, and the triple combination, respectively. Within these fractions, the proportion of cells classified as apoptotic was highest in the triple-combination group (35.39%), whereas the control group showed the lowest apoptotic fraction (5.0%) (Figure 5).

### 2.6. Intracellular Ceramide Levels

**Figure 6:**
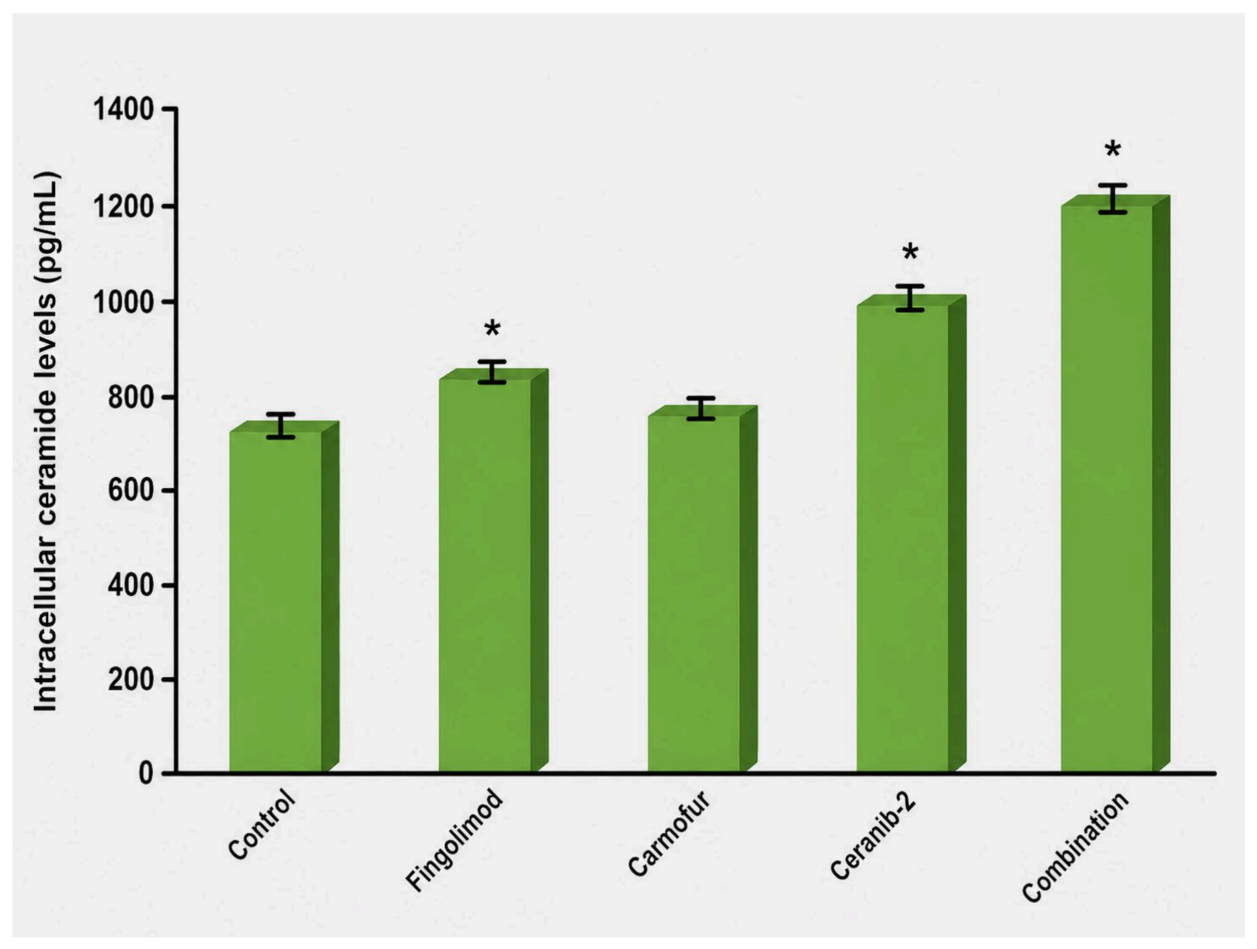
Intracellular ceramide levels in SH-SY5Y cells. Data are presented as mean ± SD from three independent experiments (*n* = 3). Statistical comparisons were performed using one-way ANOVA followed by Tukey’s post hoc test. * *p* < 0.05 versus the control group.

Intracellular ceramide concentrations measured in SH-SY5Y cells were 747.78 ± 13.17 pg/mL for the control group, 851.23 ± 7.09 pg/mL for fingolimod, 777.04 ± 12.34 pg/mL for carmofur, 995.24 ± 8.54 pg/mL for ceranib-2, and 1190.59 ± 6.63 pg/mL for the triple combination group. Intracellular ceramide levels were statistically significantly elevated (*p* < 0.05) in cells treated with fingolimod, ceranib-2, and the triple combination, compared to the control group. A modest increase in intracellular ceramide concentrations was noted in cells treated with carmofur (Figure 6).

## 3. Discussion

The present study investigated the effects of fingolimod, ceranib-2, and carmofur, administered individually and in combination, on cytotoxicity, apoptosis-associated responses, clonogenic growth, and intracellular ceramide levels in SH-SY5Y neuroblastoma cells. All three agents affected cell viability and clonogenic growth, while the triple combination produced the most pronounced changes in apoptosis-associated parameters and intracellular ceramide accumulation. However, fingolimod alone showed the lowest IC_50_ value, indicating that the triple combination did not increase cytotoxic potency on an IC_50_ basis. These findings therefore suggest that the main effect of the combined treatment may be reflected in selected apoptosis-associated responses and ceramide accumulation rather than in increased cytotoxic potency. Given the molecular and phenotypic heterogeneity reported among neuroblastoma models [7,8], the present results should be interpreted as model-specific in vitro observations and should not be generalized to neuroblastoma as a whole without validation in additional genetically distinct cell models. Fingolimod is a sphingosine-1-phosphate receptor modulator that disrupts sphingolipid signaling and induces apoptosis through mitochondrial stress and inhibition of survival pathways [25,26]. The balance between ceramide and S1P is known to regulate cancer cell survival, with ceramide generally promoting cell death and S1P supporting proliferation and survival signaling [27]. In the present study, the triple combination was associated with greater ceramide accumulation and more pronounced apoptosis-associated responses than the single-agent treatments, although it did not show greater cytotoxic potency than fingolimod on an IC_50_ basis. These observations are consistent with a shift in sphingolipid signaling toward a more pro-apoptotic state, but the current experimental design does not establish a synergistic interaction among the three compounds. Ceranib-2 is an acid ceramidase inhibitor that has been reported to increase intracellular ceramide levels and promote apoptosis in different cancer models. Previous studies showed that ceranib-2 suppresses telomerase activity and induces ceramide-associated apoptosis in non-small cell lung cancer cells [17] and promotes apoptosis in prostate cancer cells through mitochondrial dysfunction and caspase activation [28]. These observations are consistent with the reduction in cell viability and apoptosis-associated changes observed following ceranib-2 treatment in the present study. Confocal microscopy further revealed apoptosis-associated morphological alterations, including nuclear fragmentation, chromatin condensation, membrane blebbing, cell shrinkage, and cytoskeletal disruption, particularly in the triple-combination group. Such morphological alterations are recognized features of apoptotic cell death [29] and have also been reported in ceranib-2-treated cancer cells [17]. Nevertheless, the more pronounced morphological alterations observed with the triple combination should be interpreted as evidence of a combined biological effect rather than as proof of pharmacological synergy. The soft agar colony formation assay demonstrated a marked reduction in clonogenic survival in all treated groups. The triple combination produced the lowest colony count (2 ± 1 colonies), although fingolimod alone resulted in a similarly pronounced reduction (4 ± 1 colonies). Therefore, the present data support a strong inhibitory effect of both fingolimod and the triple combination on clonogenic growth, but do not establish a clear additional advantage of the combination over fingolimod alone. Colony formation reflects long-term proliferative capacity and tumorigenic potential; thus, its reduction is consistent with suppression of cellular survival and growth-related processes. Previous studies have shown that ceramide accumulation can interfere with PI3K/Akt and MAPK signaling pathways and thereby reduce proliferation and tumor growth [27,30]. Ceranib-2-based treatments have also been reported to suppress proliferation and tumorigenic potential in cancer cells [17], while fingolimod has been associated with reduced clonogenic survival through modulation of sphingolipid signaling and induction of cellular stress [26]. Taken together, these findings support the anti-clonogenic activity observed in the present SH-SY5Y model, while further experiments in additional neuroblastoma cell lines are required to determine whether this effect is reproducible across different tumor backgrounds. Annexin-V analysis showed a marked increase in early apoptotic cells in the triple-combination group compared with the single-agent treatments. This finding is consistent with enhanced apoptotic cell death following combined treatment. Ceramide accumulation has been associated with mitochondrial dysfunction, cytochrome c release, and activation of downstream caspase signaling [29], and ceranib-2 has previously been reported to increase apoptotic cell populations through ceramide-associated cellular stress and mitochondrial dysfunction [17]. In the present study, the triple combination was also associated with the highest proportion of caspase 3/7-positive cells. Caspase-3 and caspase-7 are key executioner caspases activated during the later stages of apoptosis [31], and previous studies have reported caspase activation following treatment with ceranib-2 [17,28] and fingolimod [26]. The concordance between the Annexin-V and caspase 3/7 findings therefore supports the occurrence of apoptosis in the treated SH-SY5Y cells. However, these assays alone do not establish a specific upstream apoptotic pathway. Direct assessment of additional markers, such as PARP cleavage, Bax/Bcl-2 balance, cytochrome c release, or mitochondrial membrane potential, would be required to define the molecular pathway responsible for the observed apoptotic response [32].One of the most notable findings of the present study was the increase in intracellular ceramide levels, particularly in the ceranib-2 and triple-combination groups. Ceramide is a central regulator of sphingolipid metabolism and is involved in apoptosis, autophagy, and cellular stress responses [29]. Ceranib-2 has previously been shown to increase intracellular ceramide levels and to promote cell death through modulation of sphingolipid metabolism in cancer cells [17]. Increased ceramide levels have also been associated with oxidative stress, mitochondrial dysfunction, and activation of stress-related signaling pathways such as JNK and p53 [27]. In the present study, the marked ceramide accumulation observed in the triple-combination group was accompanied by more pronounced apoptosis-associated responses. These parallel changes are consistent with an involvement of sphingolipid remodeling in the observed cellular effects, although the current experimental design does not establish a direct causal relationship between ceramide accumulation and apoptosis. Carmofur, an acid ceramidase inhibitor and antineoplastic agent, has also been reported to affect ceramide metabolism and apoptotic signaling in cancer cells [30]. Although carmofur is an acid ceramidase inhibitor, it produced only a modest and non-significant increase in total intracellular ceramide in the present study (777.04 ± 12.34 pg/mL vs. 747.78 ± 13.17 pg/mL in controls), despite its clear cytotoxic effect. This dissociation suggests that the cytotoxic activity of carmofur in SH-SY5Y cells may not be explained solely by ceramide accumulation and may also involve ceramide-independent mechanisms. In support of this possibility, carmofur has been reported to exert antiproliferative effects through additional cellular pathways, including suppression of cell-cycle progression via reduced E2F8 transcription in glioblastoma cells [23]. Taken together, the present observations support further investigation of combined modulation of ceramide- and S1P-associated pathways, but mechanistic confirmation will require additional molecular analyses and validation in other neuroblastoma models.

The effects observed with the triple treatment suggest that simultaneous modulation of multiple components of sphingolipid metabolism may influence apoptosis-associated responses in SH-SY5Y cells. However, the present data do not allow the interaction among fingolimod, ceranib-2, and carmofur to be classified as synergistic, additive, or sub-additive, because formal combination-analysis models and intermediate two-drug combinations were not included [32]. Cancer cells can maintain reduced pro-apoptotic ceramide signaling as part of mechanisms that favor survival, and inhibition of ceramide degradation has been proposed as one approach to shift this balance toward cell death [25]. Previous studies have also suggested that ceranib-2-based combinations may enhance anticancer responses through modulation of sphingolipid metabolism and apoptosis-related pathways [17]. Nevertheless, the present findings should be interpreted as preliminary observations in the SH-SY5Y model and require confirmation in additional neuroblastoma cell lines and through systematic combination analyses before broader therapeutic conclusions can be drawn. The translational relevance of the present findings should also be considered cautiously. In vitro concentrations producing biological effects cannot be assumed to correspond directly to clinically achievable drug exposures, and pharmacokinetic characteristics may differ substantially among the three compounds. Fingolimod is known to penetrate the central nervous system, whereas its systemic and CNS exposure is influenced by its pharmacokinetic properties [33]. Carmofur has a history of clinical use as an antineoplastic agent and has distinct pharmacokinetic and toxicity profiles that must be considered when evaluating its use in combination regimens [34]. In contrast, evidence regarding ceranib-2 remains predominantly preclinical, with its anticancer effects largely investigated in in vitro cancer models [35]. The present study did not evaluate pharmacokinetic interactions, clinically achievable combined exposures, or the safety and tolerability of simultaneous administration of the three compounds. Therefore, the current findings should be regarded as an initial in vitro evaluation of this combined targeting strategy rather than evidence of its clinical feasibility. Further pharmacokinetic, toxicity, and in vivo studies will be required to determine whether the observed effects can be reproduced safely under therapeutically relevant exposure conditions.

Several important limitations should be considered when interpreting the present results. First, all experiments were performed using a single neuroblastoma cell line, SH-SY5Y. Given the substantial molecular and phenotypic heterogeneity of neuroblastoma, observations obtained from this model cannot be generalized to the disease as a whole. Validation in additional genetically distinct neuroblastoma cell lines, particularly MYCN-amplified models, is therefore necessary to determine the reproducibility and broader relevance of the observed effects [7,8]. Second, although concentration-dependent cytotoxicity was assessed, the present study did not provide a comprehensive dose-by-time characterization of the individual compounds and their combination across the different biological endpoints. Systematic evaluation at multiple concentrations and time points will therefore be required to define the temporal and dose-dependent nature of these responses. Third, only the single agents and the triple combination were examined; intermediate two-compound combinations were not tested, and no formal combination-analysis model was applied. Consequently, the interaction among the three compounds cannot be classified as synergistic, additive, or sub-additive based on the present data [32]. In addition, the Annexin-V and caspase 3/7 findings support the occurrence of apoptosis but do not define the specific upstream molecular pathway involved. Additional analyses, including PARP cleavage, Bax/Bcl-2 balance, mitochondrial membrane potential, ROS production, and related signaling pathways, would be required for a more detailed mechanistic characterization. Another methodological limitation is that intracellular ceramide levels were quantified using an ELISA-based assay that measures total ceramide content but does not distinguish individual ceramide molecular species, such as C16- or C18-ceramide. Because ceramide species with different acyl-chain lengths may exert distinct biological effects [36,37], the present findings do not allow identification of the specific species associated with the observed cellular responses. Therefore, future studies incorporating LC-MS/MS-based lipidomic profiling will be necessary to characterize species-specific ceramide alterations more precisely. Another limitation is the absence of a non-tumoral or non-transformed control cell model. Because the effects of the tested compounds were evaluated only in SH-SY5Y neuroblastoma cells, the present study does not allow assessment of tumor selectivity or comparison of treatment sensitivity between malignant and non-malignant cells. Evaluation of drug responses in biologically relevant non-transformed cells is important for distinguishing tumor-selective activity from general cytotoxicity and for estimating the potential therapeutic window [38]. In addition, neuronal cell models may differ in their susceptibility to cytotoxic agents [39]. Therefore, future studies should include appropriate non-tumoral neuronal or other non-transformed control cells to better define the selectivity and translational relevance of the observed effects. Confocal microscopy was used primarily for qualitative assessment of treatment-associated morphological changes, and quantitative image-based analysis of parameters such as nuclear fragmentation, chromatin condensation, or cytoskeletal alterations was not performed. Therefore, the confocal findings should be interpreted as supportive morphological observations rather than quantitative evidence. Future studies incorporating standardized image analysis would allow more objective comparison of these morphological responses among treatment groups.

Finally, the present study was conducted entirely in vitro and did not include in vivo validation. Therefore, the effects observed in SH-SY5Y cells cannot be directly extrapolated to tumor responses in vivo, where pharmacokinetics, tissue distribution, tumor microenvironment, systemic toxicity, and drug–drug interactions may substantially influence treatment outcomes. Validation in appropriate in vivo neuroblastoma models will be necessary before the biological and translational relevance of the combined treatment can be more fully established.

Accordingly, the present findings should be regarded as preliminary in vitro observations that provide a basis for further validation rather than definitive evidence of therapeutic efficacy.

## 4. Materials and Methods

### 4.1. Culture of SH-SY5Y Cells

Human neuroblastoma SH-SY5Y (CRL-2266™ ATCC, Manassas, VA, USA) cells were cultured in Eagle’s Minimum Essential Medium (EMEM) supplemented with 10% fetal bovine serum (FBS) and 1% penicillin-streptomycin in a 37 °C incubator with 5% CO_2_. The cells were subcultured every three days. Cells in flasks at approximately 85% confluence were used for the experiments. Fingolimod (FTY720), ceranib-2, carmofur, EMEM, FBS, penicillin-streptomycin, MTT, acridine orange, and phalloidin were obtained from Sigma-Aldrich (St. Louis, MO, USA).

### 4.2. MTT Colorimetric Assay

Stock solutions of the active compounds were prepared in DMSO and diluted in culture medium so that the final DMSO concentration did not exceed 0.1% (*v*/*v*); control wells received culture medium containing the same volume of DMSO as the treated wells, so that any solvent contribution was accounted for. Cells (2 × 10^3^ per well) were seeded onto 96-well plates and incubated at 37 °C in a 5% CO_2_ atmosphere with active compound concentrations ranging from 3.125 to 100 µM for 24 h. After incubation, 20 µL of MTT solution (5 mg/mL stock in 1× PBS) was added per well and the plates were incubated for a further 2 h. The medium was then removed and the formazan crystals were dissolved in DMSO (100 µL per well); absorbance was measured at 570 nm using a Biotek HTX-Synergy microplate reader (HTX Synergy, Bio-Tek, Winooski, VT, USA). Percentage viability was calculated from the absorbance values relative to the DMSO-treated control. Three independent experiments were carried out, each with three technical replicates per concentration. For the triple combination, fingolimod, ceranib-2, and carmofur were mixed at an equal 1:1:1 ratio from their respective stock solutions. The experimentally determined IC_50_ of the triple combination was 24.85 µM in total, corresponding to approximately 8.28 µM of each compound. The same combination ratio was subsequently used in the confocal microscopy, Annexin-V, caspase 3/7, soft agar, and intracellular ceramide assays. For subsequent assays, the single agents were applied at their respective experimentally determined IC_50_ concentrations, whereas the triple combination was applied at its experimentally determined total IC_50_ concentration. This IC50-based approach was used to compare cellular responses at approximately equivalent levels of cytotoxic challenge rather than at identical absolute drug concentrations.

### 4.3. Examination of Morphological Changes Using Confocal Microscopy

Confocal microscopy was performed using 3 × 10^5^ cells per well, plated on sterilized glass coverslips in 6-well plates. Cells were cultured for 24 h at 37 °C and 5% CO_2_ under standard cell culture conditions with the determined IC_50_ concentrations of the active compounds. After incubation, cells were rinsed with PBS and fixed with glutaraldehyde using a protocol adapted from a previously reported fluorescence-imaging procedure [40]. Cells were then stained with acridine orange and FITC (fluorescein isothiocyanate)-conjugated phalloidin. Acridine orange was used to visualise nucleic acids and reveal changes in nuclear morphology and chromatin organisation, whereas FITC-conjugated phalloidin was used to stain filamentous actin and assess cytoskeletal integrity. Together, these stains allowed apoptosis-associated nuclear and cytoskeletal changes to be evaluated in the same cells [29]. The stained cells were examined and imaged using a Leica TCS-SP5 II confocal microscope with Leica confocal software version 2.00. Three independent experiments were performed (*n* = 3), and 10 fields were examined per condition.

### 4.4. Annexin-V Assessment

SH-SY5Y cells were seeded in 6-well plates at a density of 5 × 10^5^ cells per well and incubated for 24 h at 37 °C in a 5% CO_2_ environment. The cells were then treated with the IC_50_ concentrations of the active compounds and, after a further 24-h incubation, were trypsinized, collected, and rinsed in phosphate-buffered saline (PBS). In total, 100 μL of the cleaned cell suspension was mixed with 100 μL of Annexin-V reagent in a separate tube. The assay determines viable, early-apoptotic, late-apoptotic, and dead cells on the basis of Annexin-V binding to externalised phosphatidylserine together with the membrane-permeability marker included in the kit, and the percentage of viable cells was taken directly from this classification. The samples were incubated at room temperature in the dark for 15 min and then analysed using the Muse™ Cell Analyzer according to the kit instructions; 5 × 10^5^ cells were acquired per sample, and the analysis was performed in three technical replicates across three independent experiments.

### 4.5. Evaluation of Caspase 3/7 Activity

SH-SY5Y cells were initially cultured in 6-well plates (5 × 10^5^ cells per well) and then exposed to active compounds at IC_50_ concentrations for 24 h. At the end of this period, the cells were collected using trypsin, washed with PBS, and incubated with caspase 3/7 reagent. The reagent contains a substrate that becomes fluorescent upon cleavage by active caspase-3 and caspase-7, so that the fluorescence signal reflects the proportion of cells with executioner-caspase activity, while the 7-AAD counterstain identifies cells that have lost membrane integrity. Following this, 7-AAD solution was added to the samples according to the caspase 3/7 kit protocol and analysed with the Muse™ Cell Analyzer (Merck Millipore, Darmstadt, Germany); 5 × 10^5^ cells were acquired per sample, and the analysis was performed in three technical replicates across three independent experiments.

### 4.6. Soft Agar Colony Formation Assay

For the clonogenic study, SH-SY5Y cells were seeded in agar in 6-well plates at a density of 1 × 10^4^ cells per well. Cells were treated with the experimentally determined IC_50_ concentrations of fingolimod (7.84 µM), ceranib-2 (52.87 µM), and carmofur (33.41 µM), or with the triple combination at its total IC_50_ concentration of 24.85 µM (approximately 8.28 µM of each compound in a 1:1:1 ratio). Untreated cells served as the control group. Cells were cultured for 21 days, after which the resulting colonies were stained with MTT and counted. The soft agar colony formation assay was performed in three independent experiments (*n* = 3), with three technical replicates per condition. The number of colonies was presented graphically, accompanied by a visual representation of the colonies within the wells [23].

### 4.7. Analysis of Intracellular Ceramide Levels

SH-SY5Y cells were treated for 24 h with the IC50 concentrations of the individual active compounds or their triple combination. Intracellular ceramide levels were measured in cell lysates using a commercially available Human Ceramide ELISA Kit (Sunlong Biotech, Cat. No. SLD4082Hu, Hangzhou, China), according to the manufacturer’s instructions. A Sunlong Biotech ELISA-based approach has previously been used for ceramide quantification [41]. Untreated cells served as the control group. The ceramide assay was performed in three independent experiments (*n* = 3). As this ELISA-based method provides a measure of total ceramide rather than individual ceramide molecular species, species-specific changes could not be distinguished using this approach.

### 4.8. Statistical Analyses

Data from the in vitro assays are expressed as mean ± standard deviation (SD). MTT, Annexin-V, caspase 3/7, and soft agar colony-formation assays were performed in three independent experiments (*n* = 3), with three technical replicates per condition. The intracellular ceramide assay was performed in three independent experiments (*n* = 3). Concentration–response curves for the MTT assay were fitted using GraphPad Prism 9, and IC_50_ values were derived from the fitted curves. Group comparisons for the soft agar colony-formation and intracellular ceramide assays were evaluated using one-way ANOVA followed by Tukey’s post hoc test, where applicable. Annexin-V and caspase 3/7 data were presented descriptively and were not subjected to formal inferential statistical testing. Statistical significance was set at *p* < 0.05.

## 5. Conclusions

The present study shows that fingolimod, ceranib-2, and carmofur, administered individually and in combination, affect cell viability, clonogenic growth, apoptosis-associated parameters, and intracellular ceramide levels in SH-SY5Y neuroblastoma cells. The triple combination produced the most pronounced apoptosis-associated responses and intracellular ceramide accumulation and markedly suppressed clonogenic growth, although fingolimod alone showed the lowest IC_50_ value. These findings support the biological relevance of simultaneous modulation of ceramide- and S1P-associated signaling in this experimental model. However, the present findings were obtained in a single neuroblastoma cell model and should therefore be interpreted as preliminary in vitro observations. Further validation in genetically distinct neuroblastoma models, together with systematic dose–response, time–response and combination analyses, will be important to determine the reproducibility, biological significance, and broader therapeutic relevance of these findings.

## Data Availability

The original contributions presented in this study are included in the article. Further inquiries can be directed to the corresponding author.

## Abbreviations

The following abbreviations are used in this manuscript:

ASAH1	Acid ceramidase
ATCC	American Type Culture Collection
Cer	Ceramide
CerS	Ceramide synthase
CNS	Central nervous system
DMSO	Dimethyl sulfoxide
ELISA	Enzyme-linked immunosorbent assay
EMEM	Eagle’s Minimum Essential Medium
FBS	Fetal bovine serum
FTY720	Fingolimod
IC50	Half-maximal inhibitory concentration
MTT	3-(4,5-dimethylthiazol-2-yl)-2,5-diphenyltetrazolium bromide
PBS	Phosphate-buffered saline
S1P	Sphingosine-1-phosphate
SD	Standard deviation
SH-SY5Y	Human neuroblastoma cell line
7-AAD	7-Aminoactinomycin D
ANOVA	Analysis of variance
